# Immunonutrition in ERAS Protocol for Patients with Gynecologic Cancer: A Narrative Review of the Literature

**DOI:** 10.3390/life15030487

**Published:** 2025-03-18

**Authors:** Vasilios Lygizos, Dimitrios Haidopoulos, Dimitrios Efthymios Vlachos, Antonia Varthaliti, Maria Fanaki, George Daskalakis, Nikolaos Thomakos, Vasilios Pergialiotis

**Affiliations:** First Department of Obstetrics and Gynecology, Division of Gynecologic Oncology, “Alexandra” General Hospital, National and Kapodistrian University of Athens, 115 28 Athens, Greece; vlygizos@gmail.com (V.L.); dimitrioshaidopoulos@gmail.com (D.H.); vlachos.dg@gmail.com (D.E.V.); antonia.varthaliti@hotmail.com (A.V.); maria.fanaki@gmail.com (M.F.); gdaskalakis@yahoo.com (G.D.); thomakir@hotmail.com (N.T.)

**Keywords:** gynecological cancer, immunonutrition, nutritional therapy, perioperative care, enhanced recovery after surgery, ERAS, postoperative complications

## Abstract

In-hospital patients who are in the gynecologic oncology setting often suffer from malnutrition, which is one of the primary problems, the rate of which reportedly ranges from 28% to 70%. Malnutrition is a significant risk factor for immunosuppression, negatively impacting immune response and postoperative recovery capacity. At the time of the surgeries, due to their wide scope and aggressive treatments such as chemotherapy and radiotherapy, the situation becomes more serious. Those micronutrients taking part in immunonutrition, namely, arginine, omega-3 fatty acids, nucleotides, and antioxidants, have the potential to prevent inflammation, protect against infections, and promote healing after the surgery. Research has shown that immunonutrition can lower the risk of postoperative infection, promote the normal healing of wounds, and reduce the hospital stays of patients, as well as support malnutrition status during chemotherapy. This review is based on a literature search conducted in Medline, Scopus, Clinicaltrials.gov, Cochrane CENTRAL, and Google Scholar, with the last search date being November 2024. Some studies. found that perioperative immunonutrition decreases wound infections and affects some immune indexes in gynecologic oncology patients positively. However, factors such as non-compliant patients, high costs, and non-standard formulations can deter its wider use. Patient adherence drops postoperatively mainly due to nausea and decreased appetite, whereas the cost of enriched formulations acts as an economic barrier. Postoperative compliance drops from ~78% prior to surgery to ~28% due to nausea, anorexia, and chemotherapy. Additionally, cost remains a constraining factor since special formulas are 2–4 times that of normal nutrition. While immunonutrition reduces hospital stay (by ~2–3 days) and infection rate (by 25–40%), access is hindered by prohibitive initial costs and lack of insurance coverage. Approaches such as subsidized schemes, enhanced palatability, and cost–benefit analyses are required to increase adoption. In addition, the lack of standardized protocols makes the clinical community hesitant to adopt this approach. Immunonutrition is, despite these problems, still hoped to be the new adjunct to gynecologic oncology patients. In future studies, it is imperative to pay attention to the best formulations that produce the best outcomes and evaluate and implement guidelines that are based on evidence. Together, with these improvements, immunonutrition could very well be an integral part of perioperative care thus completing the process by which patients in intense treatments are benefited not only via treatment but also via quality of life.

## 1. Introduction

Malnutrition in hospitalized patients is a significant concern, documented with prevalence rates from 50 up to 66 percent in specific patient populations [1,2]. This condition is particularly critical in gynecologic oncology, where malnutrition can escalate the risk of adverse outcomes due to the severe physiological stress induced by extensive surgeries, chemotherapy, and potential radiotherapy treatments, all of which contribute to severe physiological stress, increased inflammation, and oxidative damage. Notably, malnutrition has been shown to compromise immune function and exacerbate postoperative complications, negatively impacting patient recovery and overall survival rates [2,3,4].

Patients with gynecological cancers experience increased oxidative stress and systematic inflammation, which impair the immunological function and delay healing. There is a marked increase in the levels of inflammatory markers such as C-reactive protein (CRP) and interleukins (IL-6, IL-1B, and TNF-α), delaying the time of wound healing, increasing the risk of infections, and prolonging hospital stays. There has been increasing interest in using natural substances to mediate these effects. In gynecological cancer, the change inflicted by oxidative stress is responsible for increased tumor growth and postoperative complications and enhanced chemotoxicity, therefore substantiating the need for targeted nutritional strategies.

Due to the ill effects of inflammation and oxidative stress, the idea of immunonutrition arises as a very important instrument for enhancing perioperative recovery and oncologic outcomes. Immunonutrition means using specific nutrients that modulate the immune system. Arginine promotes wound healing and improves T-cell functions. Omega-3 fatty acids help diminish pro-inflammatory cytokines and thereby boost the immune system. Glutamine prevents mucositis due to chemotherapy in that it keeps intestinal walls intact. Nucleotides cause immune cell proliferation. Antioxidants block oxidative damage. Healthcare nutrition devoted to fostering a speedy recovery after surgery and decreasing the chances of infections is now incorporated into the enhanced recovery after surgery (ERAS) protocol, thereby shortening hospital stays.

Although many previous studies have been carried out on the application of immunonutrition for surgical and critically ill patients, very few studies have focused on gynecologic oncology patients. This research is unique in that it explores the importance of immunonutrition within the ERAS framework for patients with gynecologic cancer by investigating the efficacy of immunonutrition and complementary therapies, such as antioxidants and natural bioactive compounds, to speed up recovery time and address clinically relevant issues such as patient compliance, cost-effectiveness, and formulation optimization.

Growing evidence for this approach suggests that placing immunonutrition into daily use with anti-inflammatory supplementation within standard oncologic care programs may provide a marked improvement in surgical outcomes, immune function, and quality of life for patients. However, more standard clinical studies are needed in gynecologic oncology to improve patient adherence to nutritional therapies, develop guidelines, and optimize formulations.

The relationship between malnutrition and surgical outcomes was first highlighted in a seminal 1936 study, which found a stark mortality rate of 33 percent among malnourished patients undergoing ulcer surgery, compared to just 3.5 percent in well-nourished counterparts [3]. Decades later, a 1990s prospective study of 500 patients at a teaching hospital in England revealed that 40 percent of admitted surgical patients were undernourished and experienced an average body weight loss of 5.4 percent during their hospital stay [4]. These historical insights underscore the critical need for effective nutritional support strategies.

In gynecologic oncology, particularly in ovarian cancer patients, malnutrition rates range between 28% and 70%, with malignancy-related malnutrition potentially impairing patients’ responses to surgical stress and predisposing them to surgical field infections [5]. This disease can lead to poor surgery results, and at the same time increases the risk of postoperative complications and prolonged hospital stays [1,3]. Many epidemiological studies clearly showed a strong association between malnutrition and clinical outcomes among the patients whom we are examining, who, consequently, require targeted nutritional support programs [4,5]. To counteract these challenges, the concept of immunonutrition has emerged as a promising strategy. Immunonutrition involves the administration of nutrients known to enhance immune function, such as arginine, omega-3 fatty acids, nucleotides, and glutamine, both preoperatively and postoperatively. This nutritional approach has been shown to modulate immune responses, reduce infection rates, and promote wound healing, thus supporting faster and more complication-free recoveries [5,6].

The implementation of immunonutrition in clinical settings, especially within enhanced recovery after surgery (ERAS) protocols, aims to improve surgical outcomes and reduce complications in cancer patients. However, its adoption is hampered by challenges such as determining the appropriate timing for intervention, assessing patient compliance, and managing the costs associated with high-quality nutritional supplements. Moreover, while some studies have shown clear benefits, others report mixed outcomes, necessitating further research to establish standardized guidelines and clarify the indications for nutritional support in this patient group [6,7].

In summary, integrating immunonutrition into the care of gynecologic oncology patients offers a potential avenue to enhance perioperative care and improve outcomes. However, standardization of immunonutrition in clinical practice raises several practical concerns, including heterogeneity in nutrient formulas, variability in dosing recommendations, and different patient needs in different clinical settings. The lack of a single general guideline universally accepted for immunonutrition is a barrier to its widespread use. Most of the studies use different formulas of vital nutrients such as arginine, omega-3 fatty acids, nucleotides, and glutamine, which makes it challenging to compare the results and draw firm conclusions about the most effective formula. Moreover, differences in the timing of the administration—preoperative, perioperative, or postoperative—contribute to the inconsistencies in the reported outcomes. Among the most significant barriers to standardization is the diversity of gynecologic cancer patients in terms of nutritional status, metabolic requirements, and comorbidities. The development of individualized nutrition protocols designed for specific patient profiles can be advantageous for immunonutrition to be the most effective with the least amount of risk. Moreover, the establishment of evidence-based guidelines by means of large randomized controlled trials will be essential to the most effective formulations and dosing strategies being established [5]. To tackle these difficulties, researchers and clinicians will have to unite to establish standardized guidelines from systematic reviews and meta-analyses of the available evidence. Consensus-building also requires close and coordinated professional cooperation between professional associations, e.g., the European Society for Clinical Nutrition and Metabolism (ESPEN) and the American Society for Parenteral and Enteral Nutrition (ASPEN). In addition, the regulatory bodies can consider establishing quality control mechanisms for ensuring the consistency of the commercially available immunonutrition products that can increase clinical confidence in using these products. As research continues to evolve, immunonutrition may become an integral part of therapeutic strategies aimed at managing the complex needs of gynecologic cancer patients, particularly those who are malnourished and undergoing intensive treatment regimens [7].

## 2. Methods

All studies that examined the impact of immunonutrition in the base of ERAS protocol for patients with gynecologic malignancies were considered as potentially eligible for inclusion, provided that the outcomes of interest were present. The prime objective of this review is to evaluate the impact of perioperative immunonutrition integrated into enhanced recovery after surgery (ERAS) protocols on postoperative outcomes in patients under surgical procedures for gynecologic cancers. In surgical recovery, the beneficial role of immunonutrition may reasonably be explained by adding to perioperative care through key nutritional supplementation with arginine, omega-3 fatty acids, glutamine, nucleotides, and antioxidants. Nevertheless, despite the theoretical benefits, the use of immunonutrition in gynecologic oncology is still rather erratic, and there is scant standardization in guideline documents.

To conduct a systematic study of this area of interest, we have profiled our review using the PICO method. The population consists of patients with malignancies in a gynecologic surgery setting. The intervention is defined as immunonutrition, which takes place under an ERAS protocol. In the comparison group are patients who did not receive immunonutrition but rather standard perioperative nutritional care. The types of outcomes of interest referred to were major postoperative outcomes: infection rates, wound healing, length of stay, immune response, nutritional status, and overall survival.

The search for relevant literature used this framework and thus assured a focused and informative review of the deliberately chosen evidence. The studies selected based on inclusion criteria focused on immunonutrition during an ERAS pathway in patients with gynecologic cancers compared with standard practice. The continued greater heterogeneity of nutritional formulations and clinical protocols led one to bring forth unaddressed loopholes in current literature to pave the way for future endeavors. The inclusion criteria for the review were studies that explored the role of immunonutrition within the framework of ERAS protocols for patients with gynecologic malignancy. The included studies had to have described at least one clinical outcome of interest, such as postoperative infection rates, wound healing, hospital stay, improvement in nutritional status, or overall survival. Randomized controlled trials (RCTs), prospective cohort studies, retrospective analysis, and systematic reviews were considered if they included quantitative data on immunonutrition intervention. Adult patients (≥18 years) with gynecologic cancers treated surgically with perioperative nutritional therapy were considered.

Exclusion criteria were studies that were solely on general nutritional support and did not mention immunonutrition, case reports, opinion articles, editorials, and studies without a control or comparator group. Studies evaluating non-surgical patients or those that did not report detailed outcome measures relevant to ERAS protocols were also excluded. Studies with poor data or methodological issues, such as small sample sizes or lack of statistical analysis, were also excluded. All published studies found in peer-reviewed journals were considered but not meeting abstracts or unpublished reports. We also explored the challenges, including patient compliance, costs, and lack of standardized protocols, to highlight future directions for optimizing immunonutrition interventions.

We used the Medline (1966–2024), Scopus (2004–2024), Clinicaltrials.gov (2008–2024), Cochrane Central Register of Controlled Trials CENTRAL (1999–2024), and Google Scholar (2004–2024) databases in our primary search, along with the reference lists of electronically retrieved full-text papers for articles published in the Latin alphabet, regardless of the actual language that was used. A decision to translate languages other than English, French, German, Italian, and Spanish with online translating tools was taken before the onset of the search. The search dates from 1966 up to 2024 with the date of our last search set as 30 November 2024. Our search strategy included the text words “immunonutrition; ERAS; gynecologic malignancies malnutrition; survival”. During the search, only one keyword related to special nutrition was used, specifically “immunonutrition”. Other keywords related to the other subtypes of special nutrition were omitted. 

## 3. Consequences of Malnutrition in Surgical Patients

Decreased caloric intake leads to a reduction in body mass, encompassing fat, muscle, and skin, and eventually impacting bone and visceral tissues. This loss is accompanied by weight reduction and an increase in extracellular fluid volume [8]. As an individual’s body mass diminishes, their nutritional requirements also decrease, reflecting a more efficient utilization of food and diminished cellular work capacity. However, this decrease in tissue mass combined with lower work capacity can hinder the body’s normal homeostatic responses to stressors such as surgery or critical illness [9].

Surgical stress or trauma induces a catabolic state that escalates the consumption of proteins and energy. Essential macronutrients stored in fat tissue and skeletal muscle are mobilized to support more metabolically active organs, like the liver and internal organs. This shift can prompt the onset of protein-calorie malnutrition—characterized by a deficit of 100 g of nitrogen and 10,000 kcal—within just a few days [9]. The speed at which postoperative malnutrition develops depends on the patient’s initial nutritional state, the surgery’s complexity, the extent of postoperative metabolic increase, and their ability to intake adequate calories.

Malnutrition brings about numerous adverse effects, including [8,9,10,11] heightened risk of infections, impaired wound healing, increased prevalence of pressure ulcers, bacterial overgrowth in the digestive tract, and atypical nutrient losses via feces, as summarized in Table 1.

For surgical patients, concerns center on heightened risks of postoperative infections and compromised wound healing. Malnutrition impairs immune system functionality, affecting complement activation, bacterial opsonization, and the performance of neutrophils, macrophages, and lymphocytes [8,9]. Research indicates that malnourished individuals display diminished skin reactions to Candida and lower antibody levels against various Phyto mitogens, suggesting both humoral and cell-mediated immunity are compromised [12]. Additionally, while protein-energy malnutrition is linked to slower wound healing rates, most wounds do eventually close [13,14].

Specifically, patients presenting with preoperative hypoalbuminemia, whether in isolation or coupled with chronic conditions like liver disease or heart failure, are more likely to experience postoperative organ dysfunction (affecting cardiac, pulmonary, renal, and hepatic systems), gastrointestinal bleeding, nosocomial infections, extended mechanical ventilation periods, longer ICU stays, and inpatient mortality [15]. Interestingly, a body mass index (BMI) below 20 kg/m^2^ has been associated with lower morbidity and mortality rates compared to higher BMI levels, although this finding contrasts with other research indicating that BMI alone is an inadequate measure of nutritional status and should be considered alongside other clinical indicators [16]. Despite being a popular measure of nutritional status, the accuracy of BMI as an indicator of malnutrition in gynecologic cancer patients is constrained. The inability of BMI to differentiate fat mass from lean body mass means that it continues to be an inadequate independent marker, particularly in cancer patients predisposed to cachexia or sarcopenia with normal or high BMI. Malnutrition in these patients typically presents as muscle wasting and micronutrient depletion rather than low body weight per se. The literature has shown that patients with normal BMI but reduced muscle mass have poorer surgical recovery, increased complications, and prolonged hospitalization. Therefore, further specific nutritional assessments, including bioelectrical impedance analysis (BIA), serum albumin, and muscle function tests, must also be included with BMI to appropriately evaluate malnutrition risk and optimize perioperative nutrition in gynecologic cancer patients. In a subsequent study involving 1164 patients undergoing coronary bypass graft surgery, preoperative hypoalbuminemia did not increase immediate postoperative mortality or morbidity but was a predictor of poorer long-term survival [16,17].

The first task when considering perioperative nutritional recommendations is to assess whether the patient has malnutrition. The clinical assessment of the surgical patient includes a complete history and physical examination on admission and assessment of protein status and may include other laboratory studies.

## 4. Immunonutrition: Concept and Mechanisms

Immunonutrition is the term given to the optimization of nutritional support with some nutrients known to be modulators of the immune system. Nutrients classified under this group are arginine, omega-3 fatty acids, nucleotides, and antioxidants [5]. These are added to standard nutritional formulations with the aim of promoting immune function, reducing inflammation, and supporting recovery, especially in surgical and chemotherapeutic patients.

The semi-essential amino acid arginine plays a crucial role in protein synthesis and acts as a precursor for the synthesis of nitric oxide, a molecule that further contributes to the modulation of immune responses. Some studies such as that of Zheng et al. [17] have shown augmentation in T-cell function, increased wound healing, and a lower risk of postoperative infection with the use of arginine. Therefore, the supplementation of arginine should be carefully managed, especially in critically ill patients, because excessive arginine may exacerbate certain conditions.

Omega-3 fatty acids are polyunsaturated fatty acids with high necessity for nutritional support. They have strong anti-inflammatory properties and influence immune functions by lowering the synthesis of proinflammatory cytokines and eicosanoids that contribute to the acute inflammatory response [18]. Omega-3 fatty acids are particularly important for patients with a predisposition to hyperinflammation, such as after surgery or chemotherapy. The modulation of post-traumatic and post-chemotherapeutic inflammatory reactions leads to a decrease in the rate of infections and hospital stays.

Nucleotides are the building blocks of DNA and RNA and hence are important nutritional components for cell duplication and immune activity. Because they replicate very rapidly, they play an important role in high-turnover cells, such as those in the immune system. Indeed, the clinical literature shows that nucleotide supplementation enhances gut integrity, immune function, and recovery following major surgery and chemotherapy treatment.

Antioxidants are important molecules for neutralizing free radicals, which are the byproducts of cellular metabolism that are toxic and dangerous to cells and other structures, including immune cells. Through the reduction of oxidative stress, antioxidants help maintain immune function and reduce inflammation, which is important in cancer patients facing increased oxidative stress from both the disease and its treatment.

**Figure 1:**
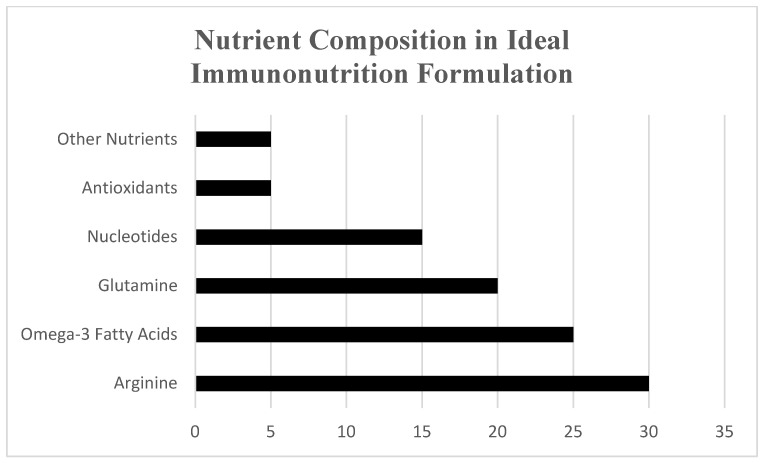
Overview of nutrient composition in immunonutrition formulations (adapted from Gupta & Senagore, [5]; Celik et al. [19]; Ay et al. [20]; Hoang et al. [21]; Zheng et al. [17].

These components of immunonutrition work together to modulate the immune system, reduce inflammation, and enhance the body’s response to cancer treatment (Table 2, Figure 1). Figure 1 provides also an overview of the nutrient composition utilized in these formulations, adapted from Gupta & Senagore [5], Celik et al. [19], Ay et al. [20], Hoang et al. [21], and Zheng et al. [17], highlighting the synergistic roles of key nutrients in reducing postoperative complications and enhancing patient recovery. In surgical patients, this happens to decrease complications after surgery, including infections, and improves healing time. In addition, during chemotherapy, these nutrients help in lowering the symptoms associated with the treatment, thus improving the treatment outcome in general. However, the efficacy of immunonutrition can vary depending on the patient’s condition, the timing and duration of the intervention, and the specific combination of nutrients used. More research is needed to optimize these interventions for different clinical scenarios.

## 5. Current Evidence on Immunonutrition in Gynecologic Oncology

Immunonutrition has gained increasing attention in the field of gynecologic oncology. This approach involves the inclusion of key nutrients such as arginine, omega-3 fatty acids, nucleotides, and antioxidants in standard nutritional formulations. Surgical intervention remains the gold standard in the treatment of gynecologic cancers, particularly ovarian and endometrial cancers, where radical procedures are often required. However, these surgeries are associated with high rates of postoperative complications due to the aggressive nature of the procedures and the fragile nutritional status of many patients from the time of diagnosis is established till surgery is performed. A study conducted by Çelik et al. [19] evaluated the effects of perioperative immunonutrition in patients undergoing gynecologic oncologic surgery. The randomized controlled trial included 50 patients who were assigned to either an immunonutrition group or a standard nutrition group. The immunonutrition group received a specialized enteral diet enriched with immune-modulating nutrients, including arginine, omega-3 fatty acids, and nucleotides, two days before and seven days after surgery. The study found that patients in the immunonutrition group had significantly lower rates of wound infections, shorter hospital stays, and improved immune parameters compared to the control group [19].

Chemotherapy is another critical component of treatment for ovarian, cervical, and endometrial cancers. However, the immunosuppressive effects of chemotherapy, along with its potential to induce severe side effects such as mucositis and myelosuppression, pose significant challenges to patient outcomes. Immunonutrition may help mitigate these effects by supporting the immune system during treatment. Research by Ay et al. [20] focused on the role of immunonutrients, specifically glutamine and omega-3 fatty acids, in patients undergoing chemoradiotherapy. The study demonstrated that supplementation with these nutrients helped maintain gut integrity, reduced the incidence of severe mucositis, and improved overall nutritional status during treatment [20]. This suggests that immunonutrition could play a critical role in supporting patients through the intensive regimens required for gynecologic cancers.

### 5.1. Omega-3 Fatty Acids

Omega-3 fatty acids, in particular EPA, DHA, and ALA, have potent anti-inflammatory and immunomodulatory effects and thus play an important role in managing chronic diseases and recovery after surgery or cancer therapy. Polyunsaturated fatty acids prevent inflammation, maintain cell membrane integrity, and may influence the growth of cancer by modulating immune responses and inhibiting pro-inflammatory mediators. Omega-3 fatty acids reduce the level of inflammatory eicosanoids produced by competitive incorporation with arachidonic acid into cellular membranes. This effect is considered to be related to a reduced type of chronic inflammation associated with cancer progression. Furthermore, omega-3 fatty acids are believed to have a direct anticancer effect in terms of their impact on gene expression, which is associated with cell proliferation, apoptosis, and tumor growth. Clinical studies also report an improvement in chemotherapy efficacy and quality of life by omega-3 intake, with mechanisms that include modulation of immune response and, to some extent, a reduction in the adverse effects of treatment [18,19]. However, despite these promising mechanisms, clinical outcomes on dietary omega-3 fatty acid intake and cancer risk have not been consistently demonstrated. Some meta-analyses have pointed toward a protective effect in case–control studies, although these could be attributed to biases in the collection of retrospective data, while prospective cohort studies have not readily supported these findings. This suggests that dietary factors in cancer prophylaxis are complex and depend on various confounders, including genes, environment, and lifestyle that modulate the observed effects of omega-3 fatty acids on cancer risk. Nonetheless, the theoretical benefits and partial demonstration of omega-3 fatty acids in reducing inflammation and likely inhibiting cancer progression, bode well for further testing, especially in well-designed clinical trials that may offer more definitive answers on this issue of potential benefit to prevent and manage cancer.

In a meta-analysis study by Hoang et al. examining the effects of omega-3 fatty acids in patients with gynecologic cancers, it was found that these nutrients not only supported the immune response but also contributed to better nutritional outcomes. Patients receiving omega-3 supplements exhibited improved serum protein levels, better maintenance of muscle mass, and a lower incidence of chemotherapy-induced cachexia [21].

### 5.2. Arginine

Another nutrient we find in immunonutrition formulas is arginine, a semi-essential amino acid, that plays a crucial role in immune function by serving as a precursor for nitric oxide synthesis. Nitric oxide is vital for various immune responses, including the activation of T cells and the modulation of macrophage activity. Arginine supplementation has been shown to enhance wound healing, reduce the risk of postoperative infections, and improve overall immune function in cancer patients. Arginine is an important amino acid mostly involved in immune functions and especially during physiological stress such as surgery. This is essential for active immune cells such as T cells and natural killer (NK) cells. A lack of perioperative arginine may undermine the recovery of the body and its immune responses as surgery stress usually depletes arginine. Studies have investigated the use of arginine in the perioperative process to reduce the negative effects of surgery on the immune system. After surgical interventions, supplementary arginine could broaden the recovery of T cells. This is an important factor for enhancing control over metastatic disease. In contrast, postpartum perioperative arginine does not always enhance NK cell effects in the first few hours post-surgery. In this case, no arginine intervention was used, but the effects were later allayed by exploiting peripheralcytes’ functional activities to secrete and produce interferon γ, releasing natural killer cells and exploiting their abilities to produce a type of intracytoplasmic cytokines. The positive effects of perioperative administration of arginine have been demonstrated in several studies, which indicate that it shortens the time of recovery of patients and reduces the incidence of postoperative complications and infectious diseases. It is also possible that the mannose and arginine enhancement of NK cell functions in vivo after surgery is useful in reducing the burden of remaining metastases [22]. Nevertheless, due to the mechanism of action of medicines such as arginine-containing medicines, it is recommended that their administration be handled with care in some patients. Although in general, L-arginine has better and safer mechanisms of action, it has also been linked with potential side effects in some patients such as nitric oxide overproduction in septic patients due to its influence on the synthesis of nitric oxide. Similarly, the metabolism of amino acids like arginine might be misregulated to impose high ammonia levels leading to the gradual deterioration in the patient’s mental state in liver failure patients, which is manifested as hepatic encephalopathy. The use of arginine-enriched immunonutrition can lead to medical problems, and, therefore, it should be employed only when the healthcare provider understands the pros and cons based on the patient’s overall clinical condition and metabolic state.

### 5.3. Nucleotides

Nucleotides, the building blocks of DNA and RNA, are essential for the rapid proliferation of immune cells. Their inclusion in immunonutrition formulations is particularly beneficial for patients undergoing surgery or chemotherapy, as these treatments can deplete the body’s nucleotide reserves, leading to compromised immune function.

According to a randomized controlled trial performed by Qin et al. investigating the effect of nucleotide supplementation during chemotherapy in patients with ovarian cancer, study participants who received nucleotide-enriched nutrition returned to health more rapidly, had lower infection rates, and showed better overall immunity [23]. Nucleotides are required for the rapid division of cells, including cells from the immune system, for example, lymphocytes. There should be sufficient provision of nucleotides during the immune response and care, which is important, especially following surgery, infection, or in immunocompromised cancer. They also have a very important function in the efficient activity of the intestinal bowel. These are thought to aid the healing and restoration of the inner surface of the intestine, which would be useful in treating recovery patients or during chemotherapy, treatments that are known to compromise gut wall integrity. They are critical in the effective generation of T cells and B cells in relation to the adaptive immune response. Their effectiveness involves not only these cells’ capacity to undergo cell division in response to immune challenges but also their capacity to create a lasting immune memory response which would be essential for resistance to pathogens over prolonged periods. This illustrates the role of nucleotides in the enhancement of the immune system.

### 5.4. Glutamine

Glutamine, while typically classified as a nonessential amino acid, becomes conditionally essential during stress, serving as a crucial precursor for nucleotide synthesis and as a vital energy source for rapidly proliferating cells, such as those in the gastrointestinal (GI) tract epithelia. It is a component of immunonutrition formulas with a fundamental role in the immune and gastrointestinal system particularly in individuals recovering from operative procedures or facing extreme ill health. It is also one of the major energy substrates for the lymphocytes and macrophages and most of the immune cells, fueling their growth and ideal activities in times of stress or infections, which is crucial after surgical procedures due to immune suppression and higher risks of both infections and complications. In addition, these cells form a critical barrier to the penetration and invasion from pathogens and xenobiotics into the body, which is of therapeutic value after surgeries in the abdomen or the use of chemotherapy drugs, which damage the gut. Furthermore, controlling the level of cytokines and synthesizing gluten helps achieve fitness by eliminating stress. In critical care settings, it has been found that additional restructuring of glutamine in these conditions has a positive effect, specifically on the disease and recovery period, helping to heal the respiratory tract or reduce chest infections after brain surgery [24]. Research has demonstrated that glutamine can improve nitrogen balance, support immune function, and reduce the incidence of infections, which are crucial for patients recovering from major surgeries. It has also been noted for its role in promoting antioxidant defense, regulating gene expression, and supporting intestinal health, which are vital for patients undergoing cancer treatments that often compromise nutritional status. Despite these benefits, the practical application of glutamine supplementation varies, with some studies showing significant improvements in patient outcomes while others indicate minimal to no impact. In critical care scenarios, supplementation of glutamine has been associated with improved patient outcomes, including reduced infection rates and enhanced recovery times, by meeting the high metabolic demands of rapidly dividing cells across both the immune system and the entire body. Its application in clinical settings, particularly as a component of immunonutrition regimens, has been shown to diminish postoperative complications, curtail hospitalization durations, and elevate survival rates, especially in critically ill and cancer-afflicted patients, thereby underscoring its pivotal role in bolstering physiological resilience and recovery in medically exigent circumstances. This variability highlights the complexity of nutritional needs during critical care and the necessity for personalized nutritional strategies to optimize patient recovery and long-term health outcomes. Therefore, while glutamine has demonstrated promise in the realm of immunonutrition in patients with gynecologic malignancies, its efficacy is unclear and needs to be investigated further to develop firm clinical guidelines and broaden its application [24,25].

## 6. Discussion

### 6.1. Immune System Cells and Immunonutrition

The direct effects of specific nutrients in immunonutrition encompass different aspects of T-cell activity, from proliferation to cytotoxicity. Such nutrients prime T-cell functions for better surveillance and responsiveness against tumor cells, which are crucial in cases of malignancies, where the ability of T cells to recognize and destroy tumor cells is most important [26,27,28]. Immunonutrition properties are attributed to its inclusion of several amino acids like arginine, an important component that improves the production of T cells in the host, which strengthens the defenses against cancer cells. Omega-3 fatty acids also modulate the potential of inflammatory responses in the microenvironment of the tumor, which affects T-cell activities through regulation further down the line in the production of suppressive factors that inhibit T-cell functionality. This allows an immune environment conducive to strong antitumor reactivity. Glutamine, as an essential ingredient in immunonutrition, serves to support T-cell function in states that are generally stressful and metabolically demanding by serving as a major energy source and enhancing antioxidant defenses. Indeed, the importance of these nutrients is clearly manifested in cancer treatment, using strategies such as neoadjuvant chemotherapy and fostering a strong T-cell response to determine the outcome of surgical interventions [28,29].

Immunonutrition helps to maintain and render T cells responsive within the unfriendly tumor microenvironment, which is harsh due to limited nutrient supplies and the presence of immunosuppressive signals. Immunonutrition not only amplifies the direct killing of tumor cells by T cells in a state of good health but also orchestrates a broader immune response involving other cell types such as natural killer cells and macrophages. This is crucial since T cells on their own often need the rest of the immune system for support in selectively killing cancer cells. T cells require substantial energy and biochemical support to proliferate, differentiate, and maintain their cytotoxic activity. Amino acids, especially arginine, are major precursors for direct T-cell proliferation through protein and molecule synthesis in cell division. Arginine induces the release of nitric oxide (NO), which is essential for the survival, activation, and killing competence of T cells toward target cells, including cancerous ones. Arginine deficiency impairs T-cell responses, so supplementation with this amino acid is crucial for boosting anti-tumor immunity [28,30,31].

In addition to amino acids, omega-3 fatty acids such as eicosapentaenoic acid (EPA) and docosahexaenoic acid (DHA) are potent modulators of the immune environment. These fatty acids alter the lipid composition of cell membranes, which is critical for receptor function and T-cell signaling. Omega-3s reduce the synthesis of pro-inflammatory cytokines such as TNF-α and IL-6, and suppressive factors like prostaglandin E2 (PGE2), which inhibit T-cell function in the tumor microenvironment. By mitigating these suppressive signals, omega-3 fatty acids create an environment more permissive for T-cell activation and increase their capacity to mount an effective response against tumor cells. Additionally, omega-3s help maintain a balance between regulatory T cells (Tregs) and effector T cells by reducing the immunosuppressive effect of Tregs while enhancing cytotoxic T-cell activity [27,29,31].

Glutamine is another crucial nutrient that serves as a vital energy source for rapidly dividing cells like T cells. During metabolic stress, such as cancer treatments like chemotherapy, T cells experience high energy demands. Glutamine supports oxidative phosphorylation and glycolysis, the two main pathways through which T cells gain energy. Moreover, glutamine is a precursor to glutathione, a key antioxidant that protects T cells from oxidative stress and damage, common in the hostile tumor microenvironment. This protective role allows T cells to function in harsh conditions, sustaining prolonged immune responses without succumbing to oxidative damage [32,33,34].

Immunonutrition not only enhances the functions of individual T cells but also supports the broader immune network by facilitating crosstalk between T cells and other immune cells, such as natural killer (NK) cells, dendritic cells, and macrophages. Immunonutrition improves T-cell viability, proliferation, and capacity to react to antigens, resulting in a well-organized immune response against cancer cells. Since T cells often rely on other immune cells to fully activate and sustain their cytotoxic functions, enhancing these interactions is vital. The increased efficiency of dendritic cells in antigen presentation, for instance, further boosts T-cell activation, while macrophages and NK cells synergize with T cells to amplify the immune response [31,32,35,36].

### 6.2. Patient Compliance

One of the greatest challenges to immunonutrition use is patient compliance, particularly in the perioperative and postoperative periods. In gynecologic oncology, where patients undergo exhaustive surgery and aggressive treatment, it is difficult to follow nutritional intervention. Hertlein et al. found in a study that preoperative compliance with immunonutrition was quite good at 78.6%, but postoperative compliance plummeted to 28.6%, primarily because of nausea, vomiting, loss of appetite, and lack of motivation—common issues after surgery. To improve compliance, interventions such as customized formulations based on the level of tolerance by the patient, alternative routes for administration (such as enteral or parenteral nutrition for non-oral-supplicant patients), and scheduled postoperative nutrition support programs ought to be undertaken. These can minimize barriers to compliance and improve the benefits of immunonutrition in rehabilitation [6]. The difficulty in maintaining compliance postoperatively underscores the need for strategies that can support patients in adhering to nutritional regimens during the most critical recovery periods (Table 3). One approach might involve integrating more palatable or alternative forms of nutrition, such as parenteral immunonutrition for patients unable to tolerate oral intake. Moreover, healthcare providers must emphasize the importance of these interventions and provide continuous support to encourage adherence. Nutrition education has the potential to play a vital role in the increased adherence rate to dietary protocols, which is crucial in optimizing patient compliance with immunonutrition interventions. Research has shown that food literacy interventions with multifaceted approaches can considerably boost self-regulation and adherence to nutritional recommendations among patients enduring intense treatment regimens. The way that patients regulate their eating behavior is the most important factor in using immunonutrition therapy effectively. Studies point out that the ability of people to self-regulate their own behavior plays an essential role in following nutritional guidelines, especially among sick and elderly patients, for example, those treated after cancer surgery [37,38].

### 6.3. Cost Considerations

The cost of immunonutrition is another critical barrier to its widespread implementation. Nutritional formulations enriched with immune-modulating components are typically more expensive than standard enteral or parenteral nutrition. This increased cost can be a significant burden for healthcare systems, especially in settings where budget constraints are a concern. Moreover, the cost may also deter patients from adhering to recommended nutritional regimens, particularly if they are required to purchase supplements out-of-pocket.

Studies evaluating the economic effect of immunonutrition emphasize balancing its upfront expense with potential financial gain due to decreased postoperative complications. While immunonutrition formulas are more expensive than standard nutrition support, they have been shown to provide significant advantages in terms of enhanced surgical outcomes, especially in minimizing infection, shorter hospital stays, and lowering overall healthcare expenses. For example, although the specialized nutritional products used in immunonutrition, such as those containing arginine, omega-3 fatty acids, and nucleotides, are significantly more expensive than standard formulations, research has shown that they are effective in improving immune system functions and thus lead to a decrease in the number of prolonged hospitalizations, costly infection control measures, and the probability of readmission, which in turn are the major factors in increasing long-term healthcare costs. Patients who use immunonutrition are less likely to develop wound infections, sepsis, and prolonged healing, which are the main causes of high healthcare costs. Also, such complications do not appear and, hence, there is a reduction in hospital stays. Moreover, there is no need for prolonged antibiotic treatment and intensive care unit admissions, which points to the real economic benefits of immunonutrition. At the same time, currently, immunonutrition is still not widely applied due to financial difficulties and especially because of conflicts with the policies of the organization.

Healthcare administrators and policymakers can successfully make accurate decisions about the implementation of immunonutrition through careful consideration of both the upfront costs and the long-term cost savings. Immunonutrition, although costly upfront, proves its benefit through the significant drop in complications and better patient results, making it a wise investment in patient care, and concurrently, improving the effectiveness of healthcare systems [6].

### 6.4. Variability in Nutritional Formulations

Another challenge is the variability in nutritional formulations. Different studies and clinical trials often use varying combinations and dosages of immune-modulating nutrients, making it difficult to standardize treatment protocols. This variability can lead to inconsistent outcomes and complicate the interpretation of study results. For instance, some studies have used formulations rich in arginine and omega-3 fatty acids, while others have included additional components such as glutamine and nucleotides. The optimal combination and dosage of these nutrients are still subjects of ongoing research, and there is no consensus on the best approach for all patients [6,7,8].

This lack of standardization can also pose a challenge for clinicians who may be unsure of how to implement immunonutrition. This may lead to hesitation in adopting these strategies, particularly in settings where the evidence of a benefit is not robust. Further research is needed to determine the most effective formulations and to develop standardized guidelines that can be easily implemented in clinical practice.

### 6.5. Conflicting Studies and Debates on Efficacy and Safety

The efficacy and safety of immunonutrition in gynecologic oncology are subjects of ongoing debate. While some studies suggest that immunonutrition can reduce postoperative complications, enhance recovery, and improve overall outcomes, others have reported mixed or inconclusive results. For example, the study by Hertlein et al. found no significant difference in the overall complication rate or length of hospital stay between malnourished ovarian cancer patients who received perioperative immunonutrition and those who did not [6]. However, there was a trend towards a reduction in infectious complications in the group receiving immunonutrition, suggesting potential benefits that may not have reached statistical significance due to the study’s limited sample size.

Conversely, some meta-analyses and systematic reviews have highlighted the potential benefits of immunonutrition, particularly in reducing postoperative infections and enhancing immune function. These studies often cite improvements in specific clinical outcomes, such as reduced length of hospital stay and lower infection rates, which are particularly relevant in high-risk surgical populations. However, the degree of benefit and the patient populations most likely to benefit from immunonutrition remain areas of active investigation [17,24].

Moreover, there are concerns about the safety of certain immune-modulating nutrients in specific populations. For example, while arginine has been shown to enhance immune function and improve wound healing, there is evidence that it may exacerbate certain conditions in critically ill patients, such as sepsis or acute respiratory distress syndrome (ARDS). This has led to caution in the use of arginine-enriched formulations in certain clinical scenarios [20].

### 6.6. Limitations

The findings of the research show the possible use of immunonutrition in patients receiving treatment for gynecologic cancer, but several major limitations to this review must be acknowledged. One major limitation has to do with the lack of large-scale RCTs proving how well immunonutrition works for these specific patients. Most of the evidence comes from small or retrospective studies, and this makes it hard to trust the evidence and advocate for the wider use of immunonutrition in general medical care.

The content and methods of immunonutrition research are also very diverse. It is difficult to draw conclusions from studies due to the plethora of disparities in nutrient content, dosage, and time of delivery, which may also be the cause of differences in the reported results. While differences between studies remain obstacles to gaining oversight of the clinical plan, this nonetheless represents even more proof of the greater need for accuracy in the efficacy criteria when the study involves more interventions. Poor patient adherence to postoperative immunonutrition treatment is one of the significant problems. Poor compliance, which can arise due to several causes such as illness, decreased appetite, and side symptoms connected with the treatment can mitigate the potential benefits of these treatments. Immunonutrition can be made more effective through overcoming such compliance roadblocks, with alternative delivery devices, patient education improvements, and formulation enhancements. Moreover, despite some research stating that immunonutrition may be able to achieve better postoperative results by reducing infection rates and thus improving recovery, the quality of the evidence is overall rather diverse. The only way to be certain about correct guidelines and the patients most likely to benefit from immunonutrition is by running more rigorous trials under tightly controlled protocols.

To summarize, though immunonutrition is not established as a universally accepted method, it can be employed in the perioperative care of gynecological cancer patients. However, we lack quality randomized clinical trials to confirm that it is safe, and the various methods to implement it and the problem with compliance make its efficacy uncertain. The most effective way to determine the efficacy of this approach would be to conduct such clinical trials in different centers, adopt a standard recipe, and provide this to all patients for the best outcome.

## 7. Conclusions

In conclusion, the role of immunonutrition in surgical and oncologic care, particularly within the field of gynecologic oncology, represents a promising if complex area of study. The concept of immunonutrition involves the strategic administration of immune-modulating nutrients such as arginine, omega-3 fatty acids, nucleotides, and antioxidants to support the immune system and eliminate the negative effects of surgery, chemotherapy, and other cancer treatments. While the theoretical framework supporting the use of immunonutrition is robust, its practical application in clinical settings presents several challenges, and the evidence surrounding its efficacy remains mixed.

Malnutrition in hospitalized patients, especially those undergoing oncologic surgeries, is a significant concern that can adversely affect patient outcomes, including recovery rates and overall survival. The prevalence of malnutrition in gynecologic oncology patients undergoing aggressive treatments like chemotherapy and surgery is particularly high, with rates ranging from 28% to 70%. The relationship between poor nutritional status and surgical outcomes has been well-documented historically and continues to be a critical factor influencing postoperative recovery. In response, the development and implementation of immunonutrition strategies aim to address these nutritional deficiencies while simultaneously enhancing immune function.

The evidence suggests that immunonutrition can offer several benefits, including improved immune responses, reduced infection rates, enhanced wound healing, and potentially shorter hospital stays. (See Table 4 for a comparative summary of key studies on immunonutrition in gynecologic oncology.). For instance, nutrients like arginine and omega-3 fatty acids have been shown to play essential roles in modulating the immune response, reducing inflammation, and promoting tissue repair. Studies, such as those conducted by Çelik et al. [19] and Ay et al. [20], have demonstrated the potential of immunonutrition to improve postoperative outcomes in gynecologic oncology patients. Specifically, these studies found that the inclusion of immune-modulating nutrients led to lower rates of wound infections, reduced incidence of chemotherapy-related complications, and improved overall nutritional status during treatment.

However, despite these promising findings, the adoption of immunonutrition in clinical practice presents many challenges. One of the most significant hurdles is patient compliance, particularly in the postoperative period when factors such as nausea, vomiting, and poor appetite can severely hinder adherence to nutritional regimens. Research has shown that while preoperative compliance with immunonutrition is relatively high, postoperative compliance often drops dramatically. This underscores the need for more palatable and accessible forms of nutrition, such as parenteral immunonutrition for patients who are unable to tolerate oral intake.

Cost is another barrier to the widespread implementation of immunonutrition. Nutritional formulations enriched with immune-modulating components tend to be more expensive than standard nutritional options. This added cost can deter both healthcare systems and patients from embracing immunonutrition, even when the potential long-term benefits, such as reduced postoperative complications and shorter hospital stays, could offset the initial financial outlay. The economic considerations surrounding immunonutrition are particularly pertinent in settings where healthcare budgets are limited, and cost-effectiveness becomes a critical factor in treatment decision-making [36,38,39].

Furthermore, the variability in nutritional formulations used in different studies complicates the standardization of immunonutrition protocols. The inconsistency in nutrient combinations and dosages makes it difficult to draw definitive conclusions about the efficacy of immunonutrition and to establish clear guidelines for clinical use. As a result, clinicians may be hesitant to adopt immunonutrition strategies without more robust and consistent evidence [39,40,41].

Another area of concern involves the safety and efficacy of certain immune-modulating nutrients in specific populations. For example, while arginine has been shown to enhance immune function in some studies, it has also been associated with adverse outcomes in critically ill patients, such as those with sepsis or acute respiratory distress syndrome (ARDS). This highlights the importance of personalized nutritional strategies that consider the individual patient’s condition, the timing and duration of the intervention, and the specific combination of nutrients being administered [41,42].

In conclusion, immunonutrition represents a promising intervention for improving surgical and chemotherapy outcomes in gynecologic oncology patients. However, its practical application is fraught with challenges, including issues related to patient compliance, cost, variability in formulations, and the need for personalized approaches. While the theoretical benefits of immunonutrition are well-supported, the evidence from clinical studies is still emerging, with some studies demonstrating clear benefits and others yielding inconclusive results. Therefore, ongoing research is essential to further explore the potential of immunonutrition and to develop standardized guidelines that can optimize its use in clinical practice. With well-designed clinical trials and a better understanding of patient-specific factors, immunonutrition could become a more integral component of perioperative and oncologic care, offering significant improvements in the quality of life and outcomes for patients undergoing intensive treatment regimens [39,42,43].

## Figures and Tables

**Table 1:** Consequences of Malnutrition in Surgical Patients.

Consequence	Description
Heightened risk of infections	Impaired immune function due to reduced neutrophil and lymphocyte activity, leading to susceptibility to nosocomial and surgical site infections.
Impaired wound healing	Protein-energy malnutrition reduces collagen synthesis and angiogenesis, delaying wound closure and increasing risk of dehiscence.
Increased prevalence of pressure ulcers	Loss of skin integrity and reduced subcutaneous fat predispose to pressure-induced injuries, particularly in immobilized patients.
Bacterial overgrowth in the digestive tract	Altered gut microbiota and reduced mucosal integrity increase the risk of translocation of pathogens, contributing to sepsis.
Atypical nutrient losses via feces	Loss of nutrients through stool exacerbates malnourishment.
Postoperative organ dysfunction	Hypoalbuminemia and nutrient deficiencies impair vital organ function, leading to complications such as renal failure, cardiac events, and hepatic insufficiency.
Extended mechanical ventilation and ICU stays	Prolonged catabolic states and infections necessitate longer durations of intensive care, delaying recovery.
Long-term mortality (linked to hypoalbuminemia)	Hypoalbuminemia and systemic malnutrition are strong predictors of reduced survival, particularly in postoperative and critically ill populations.

**Table 2:** Key Nutrients in Immunonutrition and Their Functions.

Nutrient	Biological Function	Clinical Evidence
Arginine	Precursor for nitric oxide synthesis, enhancing T-cell proliferation, macrophage activity, and wound healing.	Associated with improved T-cell responses and reduced postoperative infections in surgical patients.
Omega-3 Fatty Acids	Anti-inflammatory effects via eicosanoid modulation; improve membrane fluidity, facilitating immune cell signaling and function.	Decrease cytokines such as TNF-α and IL-6; reduce the duration of hospital stays and incidence of sepsis.
Glutamine	Provides energy for immune cells; supports glutathione synthesis for antioxidant defense; improves gut integrity during stress.	Found to reduce infections and support mucosal healing in patients undergoing chemotherapy and surgery.
Nucleotides	Essential for DNA and RNA synthesis; support rapid proliferation of immune cells, particularly lymphocytes and macrophages.	Enhance intestinal recovery post-chemotherapy and improve immune responses during perioperative periods.
Antioxidants	Scavenge free radicals to mitigate oxidative stress; support immune cell function under inflammatory conditions such as surgery and chemotherapy-induced stress.	Linked to reduced systemic inflammation and oxidative damage in patients receiving immunonutrition.

**Table 3:** Challenges in Implementing Immunonutrition.

Challenge	Description	Proposed Strategies
Patient Compliance	Nausea, vomiting, and poor appetite in postoperative periods reduce adherence to oral immunonutrition regimens.	Develop palatable or alternative formulations such as parenteral immunonutrition; provide patient-centered education.
Cost of Immunonutrition	Immune-enhancing formulations are more expensive than standard options, limiting access in resource-constrained settings.	Demonstrate cost-effectiveness by linking reduced complication rates and shorter hospital stays to economic benefits.
Variability in Formulations	Inconsistencies in nutrient compositions and dosages among studies hinder standardization and limit clinical adoption.	Conduct robust clinical trials to establish evidence-based protocols for standardized immunonutrition.
Safety in Specific Populations	Certain nutrients, such as arginine, may exacerbate conditions like sepsis or acute respiratory distress syndrome (ARDS) in critically ill patients.	Implement personalized nutrition approaches based on patient-specific risk factors, such as metabolic state and comorbidities.

**Table 4:** Comparative Summary of Key Studies on Immunonutrition in Gynecologic Oncology.

Study (Year)	Patients (N)	Infection/Complication Rates	Hospital Stay	Mortality	Compliance
Celik et al. [19]	50 (RCT)	Significantly lower in immunonutrition group vs. control (*p* < 0.05). No difference in pulmonary or urinary infections	Significantly shorter with immunonutrition (*p* < 0.05) (exact reduction ~2–3 days).	Not reported (no deaths noted).	Not reported (perioperative immunonutrition given 2 days pre- and 7 days post-op; assumed high compliance).
Chapman et al. [28]	338 (retro. cohort)	9.6% with immunonutrition vs. 33% in control (*p* = 0.049). After adjustment, immunonutrition reduced risk of serious SSI (CDC class II–III) by ~78% (OR 0.22, 95% CI 0.05–0.95, *p* = 0.044)	~3 days shorter in immunonutrition group (difference not significant)	No significant difference (no increase in deaths or major complications)	~75% of patients adhered to post-op immunonutrition regimen (few received pre-op immunonutrition in this study).
Hertlein et al. [6]	47 (28 IM vs. 19 control)	42.9% IM vs. 42.1% control (no significant difference).	Median 18 days (IM) vs. 15 days (control), no significant reduction	No difference reported (study not powered for mortality)	78.6% pre-operative intake vs. only 28.6% post-operative (major drop due to nausea, poor appetite)
Ferrero et al. [29]	~84 (42 IM vs. ~42 control)	21.4% IM vs. 42.9% control had grade II–III complications (*p* = 0.035). Notably fewer infections requiring antibiotics in IM group (1 patient) vs. control (7 patients) (reported in full text).	7.5 days IM vs. 9.2 days control on average (significantly shorter with immunonutrition, *p* = 0.009) (LOS)	No perioperative deaths reported (no difference in survival outcomes in short term).	Preoperative immunonutrition (administered ~5–7 days before surgery)–all 42 patients in IM group received the planned immunonutrition (100% compliance pre-op).
Qin et al. [23]	60 (RCT during chemo)	Not specifically reported (study focused on nutritional status during chemotherapy). Immune function: IM-supplemented group showed higher leukocyte and lymphocyte counts than control (*p* < 0.05) , suggesting fewer immune-related complications.	N/A (no hospital stay data–outpatient chemotherapy setting).	Not reported (short-term study during chemo; no deaths reported).	High–60/60 patients completed the 15-week oral nutritional supplement course (100% adherence)

Abbreviations: RCT = randomized control trial; IM = immunonutrition group; SSI = surgical site infection; CDC = center of disease control; LOS = length of stay; N/A = not applicable; Each study showed that perioperative immunonutrition tends to reduce infection rates and/or hospital stay (Celik, Chapman, Ferrero), though one trial in malnourished patients found no significant improvement (Hertlein). Importantly, patient compliance with immunonutrition is a challenge postoperatively (dropping to ~28% in one study), which can impact these outcomes. (All *p*-values shown are as reported in each study).

## Data Availability

No new data were created or analyzed in this study.
